# L1cam Is Crucial for Cell Locomotion and Terminal Translocation of the Soma in Radial Migration during Murine Corticogenesis

**DOI:** 10.1371/journal.pone.0086186

**Published:** 2014-01-28

**Authors:** Madoka Tonosaki, Kyoko Itoh, Masafumi Umekage, Tomokazu Kishimoto, Takeshi Yaoi, Vance P. Lemmon, Shinji Fushiki

**Affiliations:** 1 Department of Pathology and Applied Neurobiology, Graduate School of Medical Science, Kyoto Prefectural University of Medicine, Kyoto, Japan; 2 Department of Psychiatry, Graduate School of Medical Science, Kyoto Prefectural University of Medicine, Kyoto, Japan; 3 The Miami Project to Cure Paralysis, University of Miami School of Medicine, Lois Pope LIFE Center, Miami, Florida, United States of America; VIB & Katholieke Universiteit Leuven, Belgium

## Abstract

L1cam (L1) is a cell adhesion molecule associated with a spectrum of human neurological diseases, the most well-known being X-linked hydrocephalus. Although we recently demonstrated that L1 plays an important role in neuronal migration during cortical histogenesis, the mechanisms of delayed migration have still not been clarified. In this study, we found that cell locomotion in the intermediate zone and terminal translocation in the primitive cortical zone (PCZ) were affected by L1-knockdown (L1-KD). Time-lapse analyses revealed that L1-KD neurons produced by *in utero* electroporation of shRNA targeting L1 (L1-shRNAs) molecules showed decreased locomotion velocity in the intermediate zone, compared with control neurons. Furthermore, L1-KD neurons showed longer and more undulated leading processes during translocation through the primitive cortical zone. The curvature index, a quantitative index for curvilinearity, as well as the length of the leading process, were increased, whereas the somal movement was decreased in L1-KD neurons during terminal translocation in the PCZ. These results suggest that L1 has a role in radial migration of cortical neurons.

## Introduction

The neural cell adhesion molecule L1cam (L1) is one of the adhesion molecules expressed in the developing central and peripheral nervous system [1], [2]. L1 plays important roles in neuronal migration, axonal growth, guidance and fasciculation, neuronal survival and synaptic plasticity [1]–[5]. A member of the immunoglobulin superfamily, L1 is an integral membrane protein with six immunoglobulin (Ig)-like domains at the amino terminal end, followed by five fibronectin type III homologous repeats, a single transmembrane region, and a highly conserved cytoplasmic tail. L1 molecules bind to a number of extracellular partners, such as the proteoglycan neurocan, neuropilin, integrins, CNTN2 (axonin-1/TAG-1), and CNTN1 (contactin/F3), as well as to L1 itself in a homophilic manner [2]–[7]. It is thought that the heterophilic and homophilic interactions between L1 molecules and various ligands are required for axonal growth, pathfinding, migration, and neuronal survival during brain development.

However, very few reports have supported the concept of neuronal migration [8]. Although it was reported that cerebellar granule cell migration *in vitro* was perturbed by the addition of anti-L1 antibody [9], to date, only one study has reported on the role of L1 upon neuronal migration in cerebral cortex [10]. L1-mRNA is expressed intensely in the cortical plate from embryonic day 13.5 (E13.5) and less intensely in the intermediate zone (IZ) at E15.5. These findings suggested that L1 is involved in various functional roles in cortical development.

Previously, using a shRNA strategy combined with *in utero* electroporation, we showed that *in vivo* L1-knockdown (KD) perturbed neuronal radial migration, accompanied with alterations in the expression of some transcription factors in the cortical plate [10]. In this approach, L1-KD apparently prevents transfected cells from recognizing a guidance or migration cue, such as L1 itself, integrins or another heterophilic ligand in the surrounding milieu that act as a substrate. In the present study, we analyzed neuronal migration in the dorsal forebrain using a time-lapse method in order to gain more insight into the underlying mechanisms of perturbed migration. The results showed that L1-KD induced slowed locomotion of young neurons in the IZ and abnormal terminal translocation with aberrant leading processes in the primitive cortical zone (PCZ).

## Materials and Methods

### Animals

All of the animal experiments conducted in this study were approved by the Institutional Review Board for Biomedical Research using Animals at Kyoto Prefectural University of Medicine, and the animals were handled according to the Institutional Guidelines and Regulations. Pregnant C57BL/6J mice were purchased from CLEA Co. Ltd. (Tokyo, Japan) or SHIMIZU Laboratory Supplies Co. Ltd., Japan SLC. (Kyoto, Japan). The day a vaginal plug was detected was designated as embryonic day 0.5 (E0.5).

### shRNA Expression Plasmid

We prepared shRNA expression plasmid as previously described [10]. Briefly a shRNA targeting L1 (shRNA2, shRNA5) and a negative control shRNA (shNC) showing no homology to any of the known mammalian genes (i.e., having a *scrambled sequence*) were cloned in linearized pGeneClip^TM^ hMGFP Vector (Promega).

The shRNA sequence is as follows.

anti-L1 shRNA (shRNA2 and shRNA5):

shRNA2; AGCCTTACCAGAAGGGAAAGT (location 3406–3426)

shRNA5; GTGCTTCAGGATGAACGATTT (location 1652–1672)

scramble shRNA (shNC); GGAATCTCATTCGATGCATAC

Detailed materials and methods were referred to in the Supplementary materials (Text S1). L1cam mRNA was efficiently downregulated in Neuro2a cells at 24 hours after transfection of shRNAs (Figure S1). The L1cam protein was efficiently downregulated in Neuro2a cells at 72 hours and 96 hours after transfection, which was confirmed by Western blot analyses (Figure S2) and immunocytochemistry (Figure S3). Finally we confirmed the disrupted radial migration when cortical neurons were transfected with shRNA targeting L1: shRNA2 and new form of shRNA; shRNA5, using *in utero* electroporation (Figure S4). Our additional experiments, as mentioned above, would strongly favor the concept that the affected phenotype induced by shRNA2 and shRNA5 did indicate *bona fide* effects of the L1-knockdown, but not off-target effects. We used shRNA2 for L1-knockdown in the following experiments.

### *In Utero* electroporation (IUE)

Plasmid DNA was prepared using an Endotoxin Free Plasmid Kit (NucleoBond Xtra Maxi EF, MACHEREY-NAGEL). Pregnant mice were anesthetized by intraperitoneal injection of Ketamine (ketalar® 50 mg/ml, Daiichi-Sankyo, Japan), Xylazine (selactar® 2%, Bayer, Germany) and saline cocktail (5/3/40 v/v/v 9.75 ml/kg bodyweight). After disinfection with 70% ethanol, a 2-cm midline laparotomy was performed, and the uterine horns were resected. In order to conduct the plasmid DNA microinjection, 0.85-mm internal diameter glass capillary tubes (HEMATOCRIT Capillary tubes, NICHIDEN RIKA GLASS Co,. Ltd., Hyogo, Japan) were employed, using a micropipette puller PB-7 (Narishige, Tokyo, Japan). After the glass capillary tubes were pulled out, the pipettes were broken at approximately 70–80 μm on the external diameter (at ca.1.0 cm from the shoulder of the pipette) by pinching with forceps, and then ground to make a 30° bevel. One μl of plasmid DNA solution (1.0 mg/ml) in endotoxin free TE buffer, with 0.005% trypan blue added to aid targeting, was injected into the lateral ventricles using a mouth-controlled pipette system (Drummond Scientific, Broomall, Pennsylvania) [11]. The embryo (E13.5) in the uterus was placed between tweezer-type electrodes, which had disc electrodes of 5 mm in diameter at the tip (NEPA GENE CO., LTD., Chiba, Japan). Electronic pulses (40V, 50 ms) were charged five times at intervals of 950 ms with an electroporater CUY-21 (Nepagene, Chiba, Japan). After the electroporation procedure, the dams were sutured at the abdomen, and the pregnancy was allowed to continue.

### Slice preparation and time-lapse observations

Embryos were harvested after 1 or 2 days, i.e., at E14.5 or E15.5. The brains were removed and transferred into a solution of 1.2% agarose (AgaroseL, Nippongene, Japan) in phosphate-buffered saline (PBS, pH 7.4) at 37°C and the agar was subsequently hardened on ice for 5 minutes. Each brain was coronally cut at 200 μm with a Vibratome (VT1000, Leica Biosystems, USA).

The following procedures were similar to those reported previously [10], [12]. The slices were gently transferred with 400 μl cold Hanks' Balanced salt solution (HBSS) onto the center of the 35 mm glass bottom dish (Matsunami glass Co,. Ltd., Japan) using disposable transfer pipettes with cut tips, then 400 μl of collagen solution prepared according to the manufacturer's protocol (Cellmatrix I-A, Nitta Gelatin, Tokyo, Japan) were added. After reducing the volume down to 200 μl, the slice-embedded masses were hardened in a 5% CO_2_ incubator at 37°C for 10 minutes, and kept in Neurobasal medium supplemented with B27 (Invitrogen, Japan), 0.5 mM L-glutamine, and antibiotics.

The slices were observed with a laser confocal scanning microscope with epifluorescence, as well as differential interference contrast (DIC) optics (ECLIPSE Ti-E, Nikon, Japan). Fluorescent microscopic images were obtained sequentially at 10-minute intervals for 24–72 hours. Approximately 20 *Z*-section images were acquired, and all focal planes were merged using EZ-C1 software. The migrating neurons were traced and analyzed using NIH ImageJ software and the Manual Tracking plugin.

### Velocity analysis

The images acquired were analyzed to trace the neuronal migration and the velocity of the migration was measured with EZ-C1 software and the NIH ImageJ plugin (Manual Tracking). The position of the center of the soma was plotted in each image, and displacement of the soma was defined by the difference in location between the starting point and the end point. The locations were expressed as the *x* and *y* values for each cell. The distances between the starting point (*x*_1_, *y*_1_) and the end point (*x*_2_, *y*_2_) were derived using the Pythagorean Theorem; the square root of the quantity of (*x*_2_ – *x*_1_)^2^ + (*y*_2_ – *y*_1_)^2^. The velocity was defined as the distance between starting and end points divided by time over the observation period [13].

### Curvature Index

The curvature index was defined as “the curvilinear length” in micrometers of a leading process divided by “the linear distance” between the ends of the leading process [14], [15]. The following analysis was performed with the NIH ImageJ Segmented Line or Multi-point Tool. “The curvilinear length” was defined as the distance traced from the apex of the soma to the tip of the leading process for a neuron in the upper cortical plate (CP). “The linear distance” was defined as the distance in a straight line between the apex of the soma and the tip of the leading process for the same neuron. Data were collected in one flattened image in which all focal planes, approximately 20 *Z*-section images, were merged.

## Results

### L1cam plays a role for radial migration of cortical neurons in IZ

**Figure 1:**
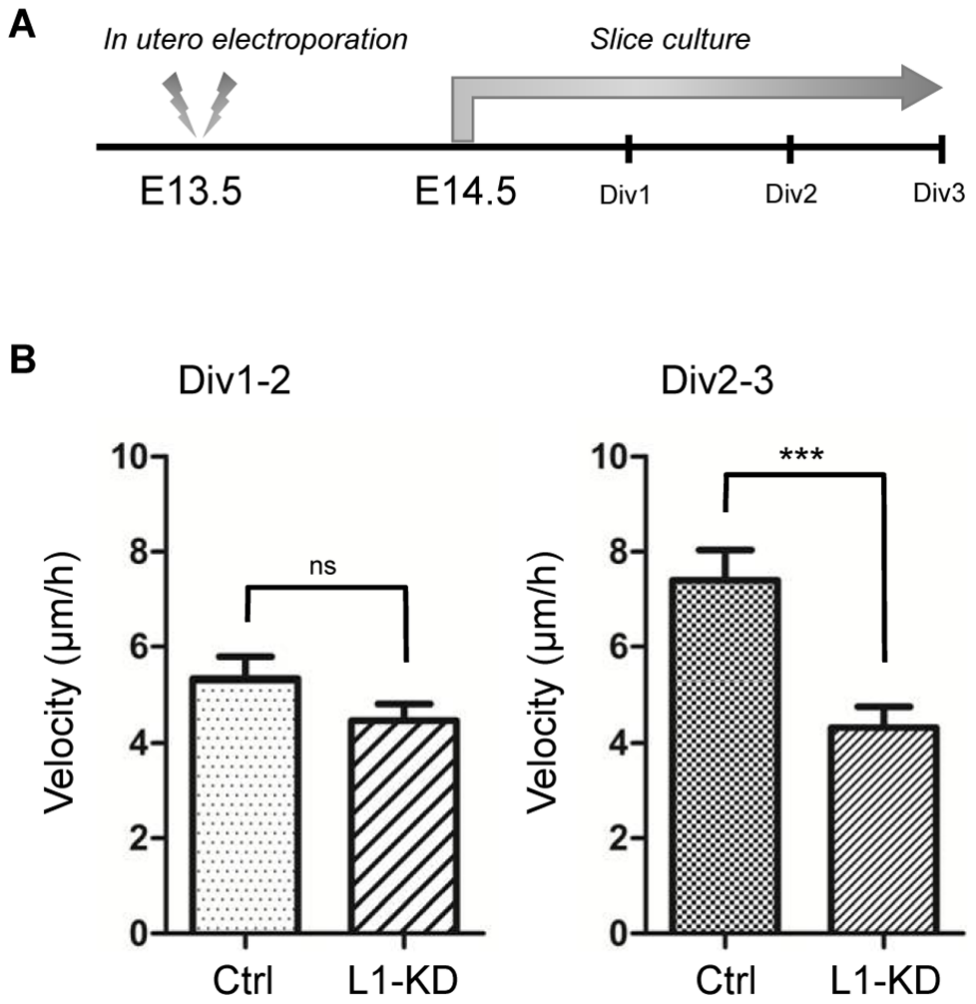
Velocity of migrating neurons in the IZ. (A) Illustration of the strategy of *in utero* electroporation and slice culture. (B) Average velocity of migrating neurons in the IZ from Div1 to Div2 or Div2 to Div3. Data were presented as Mean ± SEM, *** *P*<0.005, unpaired Student's *t*-test compared with the control (ctrl).

A large number of control neurons, which were transfected with a scrambled (non-targeting) shRNA:shNC, migrated into the IZ at E14.5, one day after the *in utero* electroporation, and then reached a point underneath the CP at E15.5. In contrast, L1-KD neurons mostly stayed in IZ at E15.5. Thus, in order to analyze the dynamic radial migration in IZ, we observed cultured slices from E14.5 to E17.5 (Fig. 1A). Almost all of the neurons stayed in the ventricular zone (VZ) or the subventricular zone (SVZ) during the first 24 hours, and then both control and L1-KD neurons began to migrate into the IZ during the next 24 hours (Div. 1–2). Although no apparent differences in the morphology were observed, the migration velocity of L1-KD neurons tended to decrease, compared with the control neurons (control: 5.32±0.46 μm/hr (n = 45 cells from 8 slices), L1-KD: 4.48±0.35 μm/hr (n = 40 cells from 7 slices); mean ± SEM, no significant difference, unpaired Student's *t-*test) (Fig. 1B). Interestingly, during the final 24 hours (Div. 2–3), L1-KD neurons migrated significantly slower than the control neurons (control: 7.39±0.64 μm/hr (n = 42 cells from 6 slices), L1-KD: 4.33±0.48 μm/hr (n = 33 cells from 7 slices); mean ± SEM, *P* = 0.004, unpaired Student's *t-*test) (Fig. 1B, movie S1 movie S2). These results suggested that L1cam played an important role in cell motility during radial migration of cortical neurons in the IZ.

### The leading processes of the L1-KD neurons were longer with aberrant morphology

**Figure 2:**
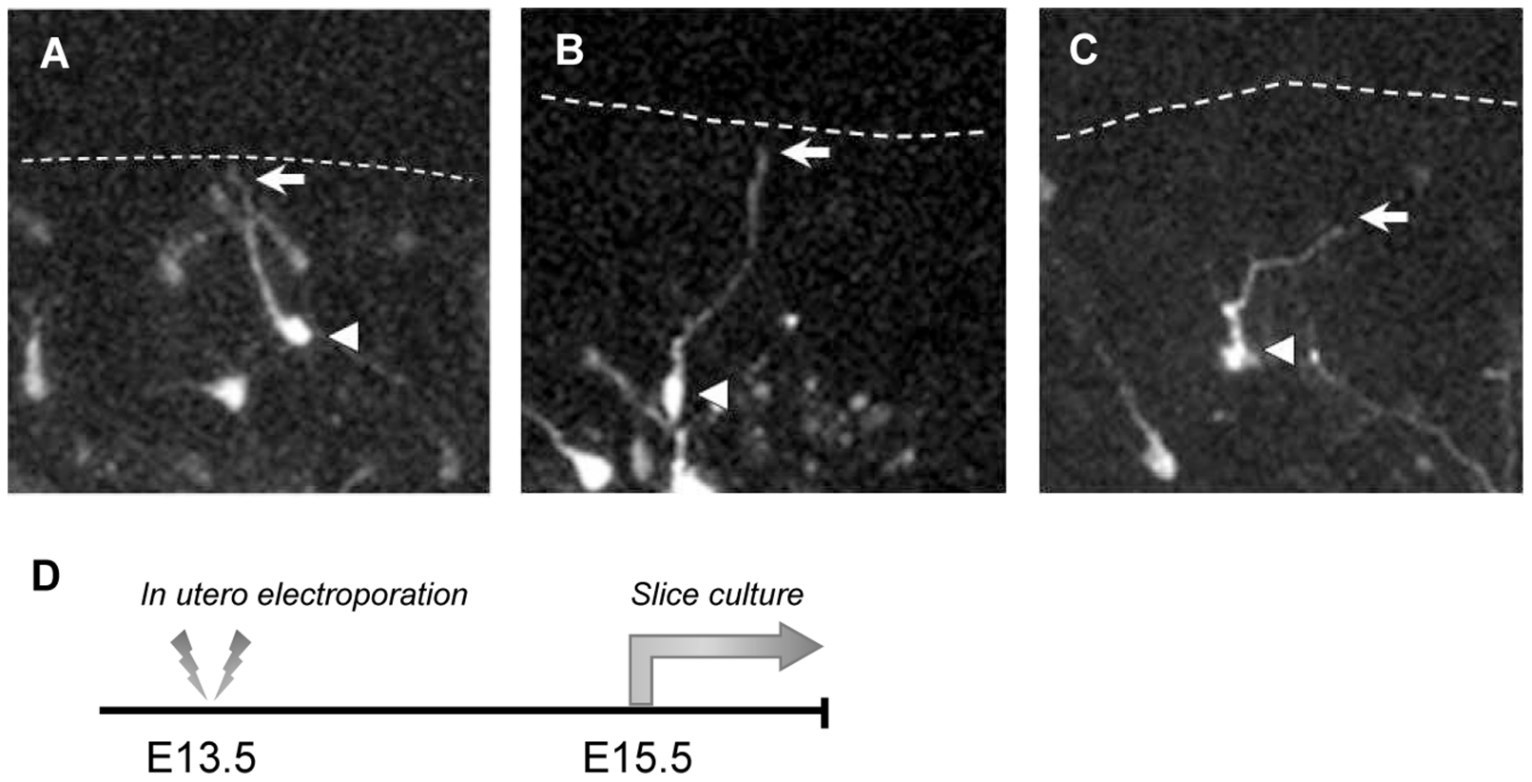
Representative morphologies of the neurons in the upper CP. (A) The leading process of a control neuron was nearly straight as it extended to the MZ. (B,C) Some L1-KD neurons had undulating processes and others could not reach the MZ, along with aberrant morphology of the leading process. The dotted line indicates the top of the cerebral cortex. The arrow indicates an edge of the leading process, and the arrowhead indicates a soma of a neuron.

The L1-KD neurons in the upper CP showed an unusual morphology, although there were no significant differences in the morphology or migration speed in the deeper CP, compared with the control neurons (data not shown). In order to assess this phenomenon, cultured slices were prepared at E15.5, two days after the *in utero* electroporation, when most of the electroporated cells were just migrating toward the pial surface in the PCZ (Fig. 2D). Although the leading processes of the control neurons extended linearly to reach the pia (Fig. 2A), the leading processes of the L1-KD neurons were undulating and some of them did not reach the pia (Fig. 2B, C). The mean length of the leading processes in L1-KD neurons (60.62±5.66 μm (n = 11)) was significantly longer than that of the control neurons (35.78±2.49 μm (n = 13); mean ± SEM, *P*<0.0001, unpaired Student's *t*-test).

In order to analyze the aberrant morphology of the leading processes of the L1-KD neurons, we compared the extent of undulation by the curvature index. The mean curvature index of the leading processes in the L1-KD neurons was 1.073±0.026 (n = 11), whereas that of the leading processes in the control neurons was 1.019±0.006 (n = 13), showing a significantly higher curvature index in the L1-KD neurons (*P = *0.0431, unpaired Student's *t*-test). These observations indicate that the leading process of a L1-KD neuron tends to be longer and more undulated than that of a control neuron. It is likely, therefore, that the process of terminal translocation is affected by L1-KD.

### The leading processes of the L1-KD neurons were highly undulated during terminal translocation

**Figure 3:**
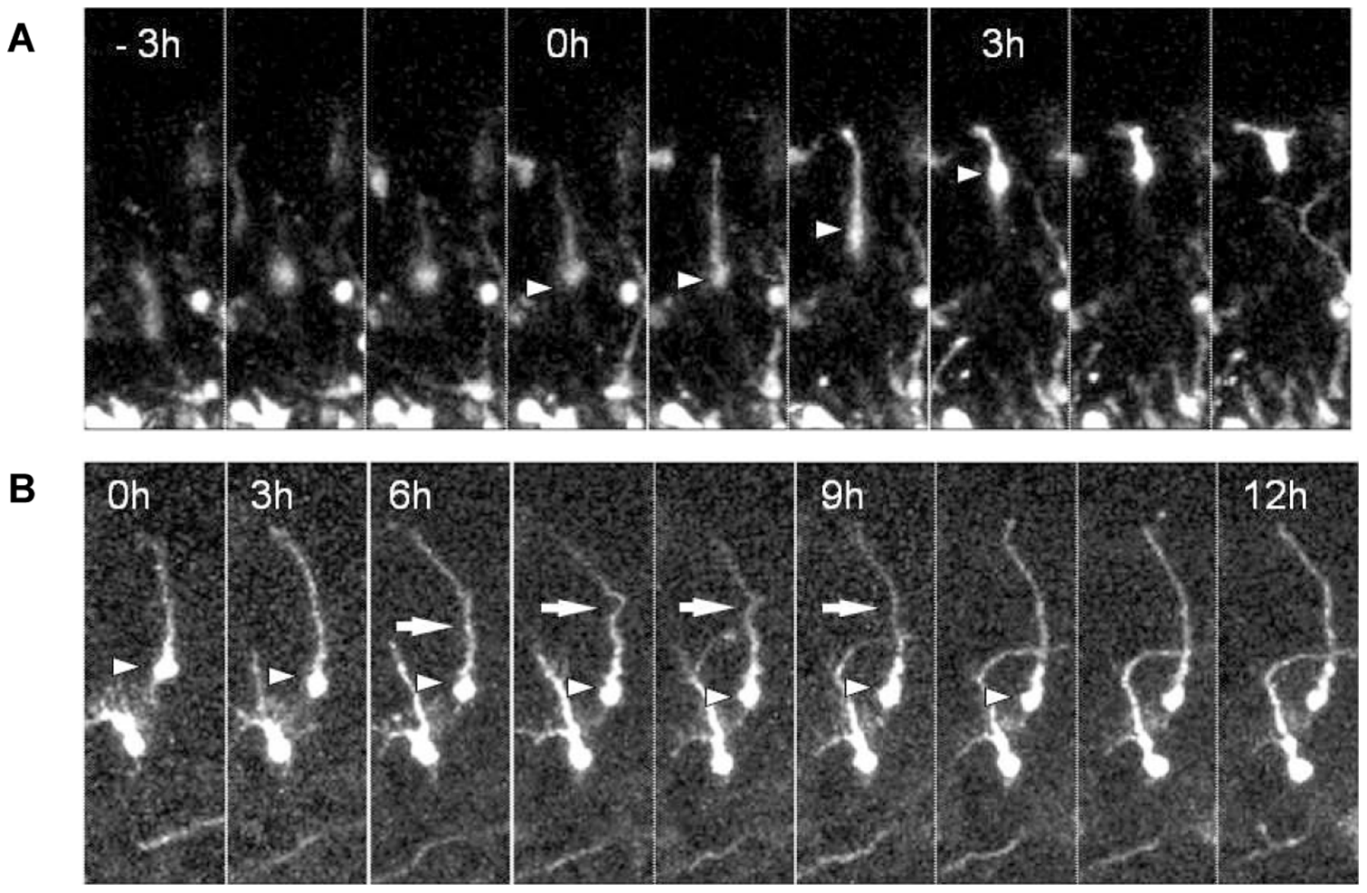
The sequential morphological changes in a leading process. (A) The leading process of a control neuron in the upper CP that was near the pia was shortened in a smooth fashion as the soma migrated towards the pia. (B) On the other hand, the leading process of a L1-KD neuron did not properly retract and was transiently undulated. Displayed on the top of the frame, the time 0 h was considered the time that the tip of the leading process first reached the pia. The arrowhead indicates the soma of a neuron, and the arrow indicates the abnormally undulating part of the leading process.

In order to gain more insight into the stage when the L1-KD neurons were affected, we focused on sequential morphological changes of the leading processes in contact with the pia during terminal translocation. The cortical slices prepared at E15.5 (2 days post-electroporation) were cultured under time-lapse microscopy for up to 24 hours. The time-lapse observations showed that most of the control neurons demonstrated continuous translocation of the cell body toward the pial surface. The leading processes of the control neurons were straight and shortened in a smooth process along with somal translocation (Fig. 3A, movie S3). However, the majority of the L1-KD neurons migrated with undulated long leading processes and some of the neurons transiently ceased migration (Fig. 3B, movie S4). The mean time required for terminal translocation of the control neurons or L1-KD neurons was 4.01±0.64 hours (n = 19) or 6.3±1.58 hours (n = 11), respectively. The time for terminal translocation tended to be longer in L1-KD neurons, compared with that in the control neurons, however; the difference did not reach the level of statistical significance. On the other hand, some L1-KD neurons did not finish terminal translocation within the observation period and settled in aberrant positions in the deeper CP with tangentially-directed leading processes (movie S3).

**Figure 4:**
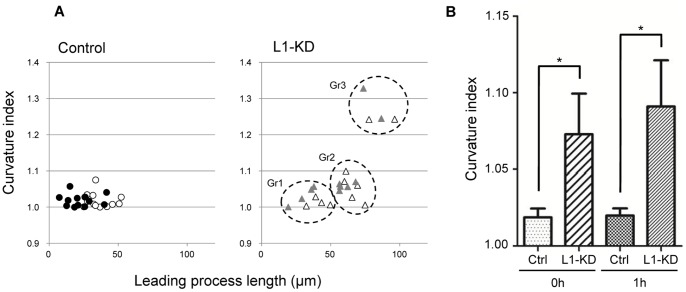
Correlation between the leading process length and the curvature index. (A) The open circles (○) indicate control neurons at 0 h. The black filled circles (•) indicate control neurons at 1 h. The black open triangles (▵) indicate L1-KD neurons at 0 h. The gray filled triangles (▴) indicate L1-KD neurons at 1 h. (B) The curvature index of the leading processes of the L1-KD neurons was larger than that of the control neurons. Data were presented as Mean ± SEM, *P<0.05, unpaired Student's *t*-test.

In order to evaluate the precise morphological changes of the leading processes during terminal translocation, we focused on the leading processes when they reached the pia mater initially (designated as 0 h), and compared those leading processes to neurons 1 h after contact (1 h). In Fig. 4, we show the relationship between the length and the curvature index of the leading processes of the control and the L1-KD neurons. The length of the control and the L1-KD neurons were 35.78±2.45 μm (n = 13) at 0 h, then 22.69±2.80 μm (n = 13) at 1 h, and 60.62±5.66 μm (n = 11) at 0 h, then 52.74±6.12 μm (n = 11) at 1 h, respectively. The length of the control neurons was shorter at 1 h, compared with the length at 0 h, while the curvature index showed little change between 0 and 1 h (*P* = 0.7870, paired Student's *t-*test) (Fig. 4). L1-KD neurons were classified into three groups according to the data on curvature index and the process length: Group 1, showing a pattern similar to that observed in the control neurons; Group 2, the intermediate between Group 1 and Group 3, and Group 3, showing a process length 2 times longer than that of Group 1, and with a higher curvature index (Fig. 4). In each group, the lengths of the leading processes of the L1-KD neurons were shortened at 1 h, compared to the lengths at 0 h, but the curvature index showed only a little change between 0 and 1 h. However, the mean curvature index of the L1-KD neurons was significantly higher at 0 h, as well as at 1 h, when compared with that of the control neurons (Fig. 4B), suggesting that the leading processes of the L1-KD neurons stayed curvilinear throughout the period of terminal translocation.

**Figure 5:**
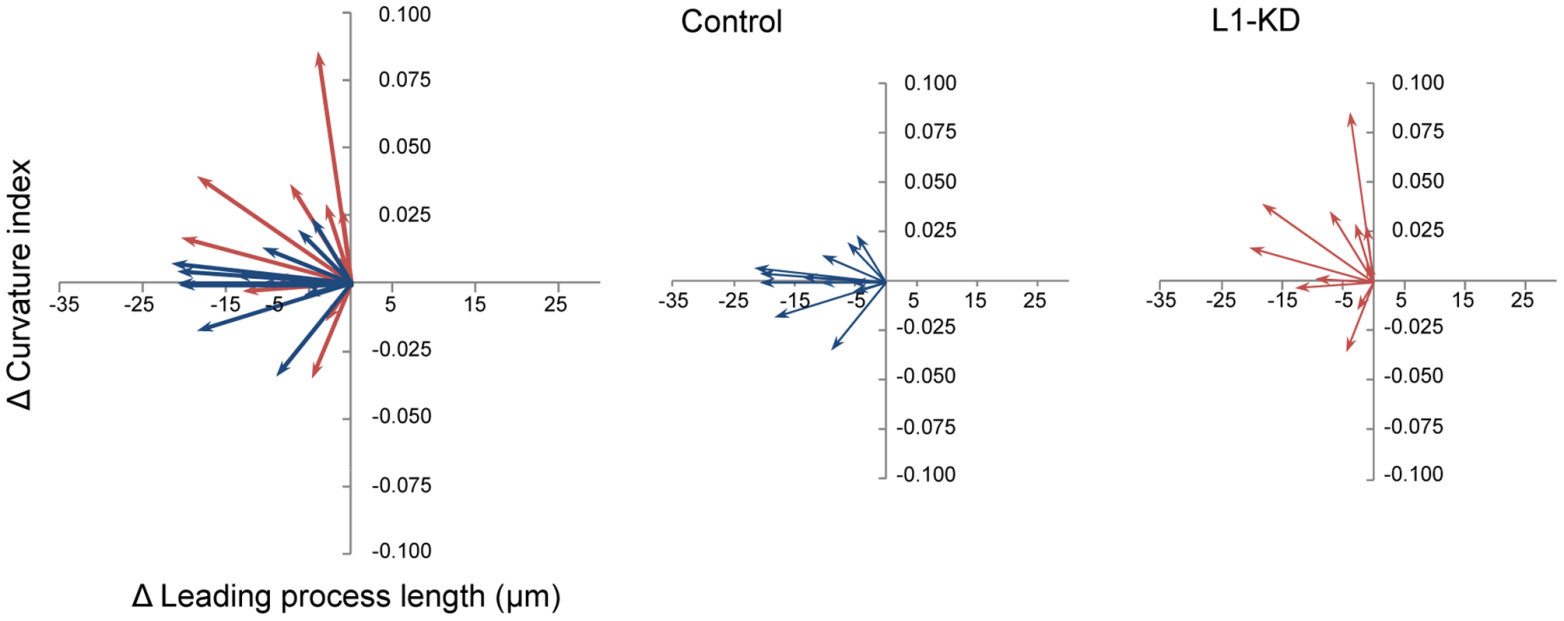
Relationships between the Δ Leading process length and the Δ Curvature index. The Δ Leading process length of both the control and the L1-KD neurons decreased by almost the same degree. In contrast, the Δ Curvature index of the L1-KD neurons increased, while the control neurons did not show much change.

Fig. 5 shows the relationship between the Δ Leading process length and Δ Curvature index. The mean length of the Δ Leading processes of the L1-KD neurons was almost the same as that of the control neurons. In contrast, the Δ Curvature index of the L1-KD neurons tended to increase more than that of the control neurons. The mean Δ Curvature index of the L1-KD neurons increased approximately 15 times more than that of the control neurons (control: 0.11% increase, L1-KD: 1.66% increase). Thus, these results suggested that L1 might be required for proper morphological changes during the early phase of terminal translocation.

### L1-KD affects proper terminal translocation

**Figure 6:**
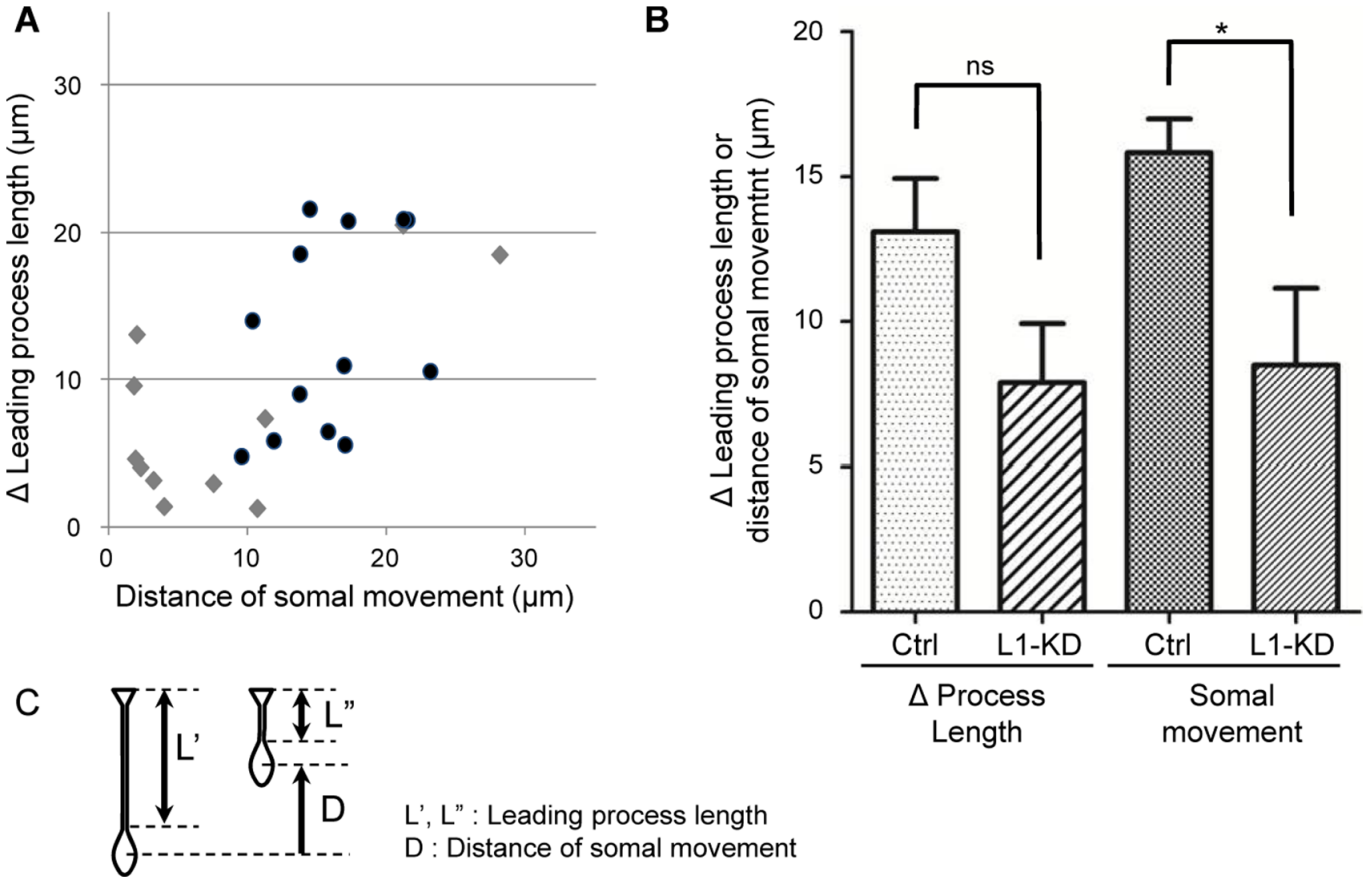
The relationship between the distance of the somal translocation and the Δ Leading process length. (A) This scatter plot shows the relationship between the distance of the somal translocation and the Δ Leading process length. The black filled circles (•) indicate control neurons. The gray filled diamonds (♦) indicate L1-KD neurons. (B) The Δ Leading process length and the distance of the somal translocation in the L1-KD was lower than that of the control neurons. Data were presented as Mean ± SEM, *p<0.05, unpaired Student's *t*-test.

We further evaluated the distance of the somal translocation in the PCZ. The *x*-axis value was the distance of each somal movement, while the *y*-axis value was the shortened length (Δ Leading process length) of its leading process, as shown in Fig. 6A. The distance of the somal translocation was defined as the linear distance between the somal position at the starting time point (0 h) and one hour later (1 h) during observation. The Δ Leading process length was defined as the difference between the process length observed at the same time points. The *x-y* distributions for a sample population of control and L1-KD neurons in the scatter plots were illustrated in Fig. 6A. Both the distance of the somal translocation and the Δ Leading process length were lower and the distribution was apparently closer to the starting point in L1-KD neurons than those observed in the control neurons. The distance of the somal translocation was significantly lower in the L1-KD neurons, compared with the control neurons (Fig. 6B). These findings suggested that the soma of the L1-KD neurons did not move efficiently during terminal translocation.

## Discussion

Recently, we demonstrated that L1cam-downregulation perturbed neuronal migration in developing murine cerebral cortex [10]. However, the mechanisms involved in the disturbance of the migration of neurons by L1-KD remain unknown. In order to elucidate which of the cellular components required for radial migration were disrupted by L1-KD, we performed time-lapse observations of the L1-KD neurons using *in utero* electroporation of L1-shRNA at E13.5.

Our time-lapse analyses revealed that the locomotion of the L1-KD neurons was significantly decreased in the IZ, which would underlie our previous findings that significantly fewer L1-KD neurons migrated into the cortical plate at E16.5, compared with the control neurons [10]. It is expected that homophilic L1-L1 interactions both in the *trans* and perhaps in the *cis*, as well as heterophilic binding of L1 to its ligands would be lost in L1-KD neurons, because full-length L1 is deficient in the cell membrane of those neurons. It is thought that these homophilic and heterophilic interactions play critical roles in the developing brain by inducing sustained activation of ERK1/2, leading to an acceleration of cell migration and L1-dependent gene expression [1]–[5], [16].

The leading processes of the L1-KD neurons were significantly longer and undulated in the upper CP. It has been reported that integrins, particularly integrin alpha3-beta1 or alpha5-beta1, seemed to mediate the interaction between the tip of the leading process and the substrate, at least in the cerebral cortex [17]–[20]. In regard to L1-KD neurons, it is expected that the interactions of the actin/ERM/ankyrin network are perturbed due to the L1 deficiency, which might lead to reduced activity in the protrusion and retraction of the leading process. In addition, a variety of cues from neighboring cells and the extracellular matrix, including L1-itself, integrins, CNTN-1 and CNTN-2, as well as proteoglycans, can not have the expected result in L1-KD neurons, as L1-dependent intracellular signaling would be ineffective. These intracellular signaling cues include activation of kinases, such as MAPK, ERK, and Src, and NF-κB pathway and gene regulation [1]–[3], [21], [22]. Although these signaling mechanisms have been revealed in studies on tumor invasiveness and metastasis associated with aberrant L1-upregulation [16], [23], some might also be involved in the developing nervous system, since there is a degree of similarity between cellular events during development and cancer progression.

During the initial phase of terminal translocation, control neurons moved quickly into the PCZ associated with the smooth retraction of the leading process. However, in L1-KD neurons, the soma remained unmoved with a longer leading process. As previously reported [24], [25], the soma of neurons moves toward the marginal zone (MZ) along with a shortening of the leading process. Cell migration involves a coordinated cycle of plasma membrane protrusion at the leading edge, adhesion site formation under the protrusion, disruption of older adhesion sites at the cell rear, and cytoskeleton contraction against adhesions to yield cell somal movement. Protrusion is thought to result from actin filament (F-actin) polymerization against the plasma membrane, with the polymerization rate regulated by the rate of monomer addition to the fast growing (“barbed”) ends of filaments [26]. When dynamic membranous expression occurs, endocytosis and recycling of L1 at the leading edge are lost [5], the adhesion formation and extension of the leading edge, disruption of the rear adhesion, and the following process growth and somal movement might be sequentially affected. Our results are also consistent with the concept that L1-signaling is a prerequisite to the retraction of the leading process and somal translocation of cortical neurons *in vivo*, although the detailed molecular mechanisms remain unknown. We showed that L1-KD neurons can be classified into three groups according to the data on the curvature index and the process length. It is anticipated that these differences might reflect the extent of L1-KD in each cell, and/or the paracrine cue from heterophilic L1-integrin interactions.

Since studies have shown that knock-in mice in which the sixth Ig domain of L1 was deleted (L1-6D) [27], [28] show severe hydrocephalus with thin cerebral cortices, and a hypoplastic, but a normally projected corticospinal tract on the C57BL/6J background, partly similar to conventional L1-KO mice [29]–[31]. In L1-dependent signaling, L1 can be cleaved by metalloproteases (MMPs) and the shed, soluble L1 forms bind to integrins, receptor type tyrosine kinases (RTKs) and L1 on the surface of the same, or neighboring cells, thereby leading to ERK1/2 activation [16], [32]. It has also been shown that the L1-cytoplasmic domain (L1-CD) is important for L1-mediated nuclear signaling and the gene regulation required for cell migration. L1-CD contains sub-domains that differ in their binding potential, and the juxtamembrane domain is critical for binding ezrin/radixin/moesin (ERM), the ankyrin binding domain and the Ran binding protein M (RanBPM) domain. The association of various partners with some of these domains can also activate ERK, thereby promoting cell motility and gene regulation through ERK pathways.

The mechanism whereby abnormal radial migration results in hydrocephalus when L1cam is deficient is still not clear. In L1-6D mice, cerebral cortex showed a decreased number of neurons and a reduced amount of subcortical white matter associated with hydrocephalus. It is tempting to speculate that affected radial migration might subsequently induce cell loss in the cerebral cortex, which lost appropriate connections with subcortical or cortical projections due to the time lag between the migrating L1-KD neurons and the arrival of the incoming fibers. Further studies are required to evaluate this hypothesis.

**Figure 7:**
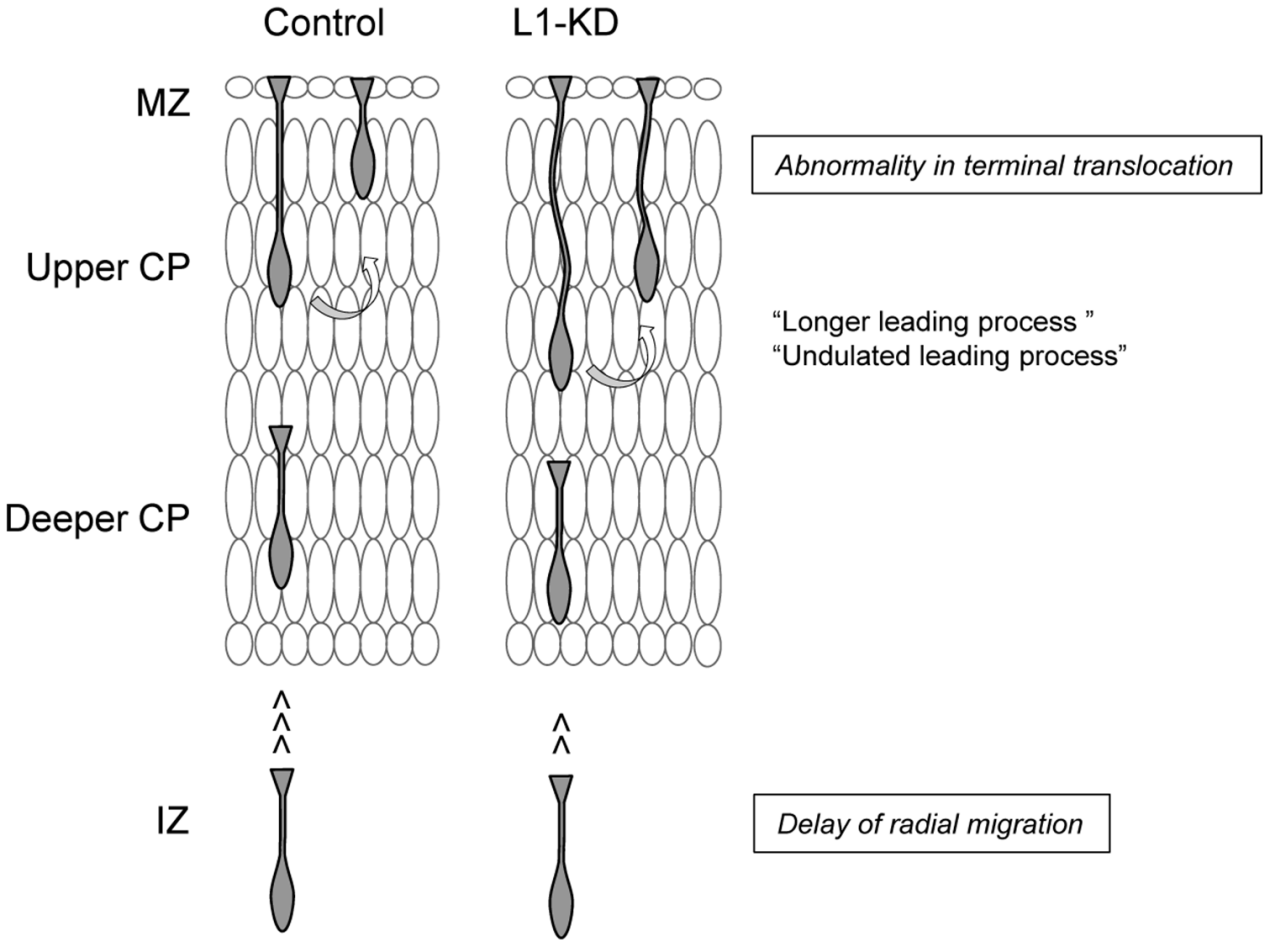
A model of the migratory behavior of L1-KD neurons. The migration velocity of L1-KD neurons decreased in the IZ, which resulted in delayed radial migration. The L1-KD neurons showed longer and undulated leading processes in the upper cortical plate and the retraction of leading processes, along with somal translocation, were disrupted during the terminal translocation of the soma in the PCZ.

Finally, we propose the schema of a novel role of L1 in cortical migration during development (Fig. 7). L1-KD induced a delay of radial migration in the IZ and an abnormal terminal translocation of the soma with inappropriately long and undulated leading processes. These results suggest that L1 is important in cell locomotion in the IZ and proper retraction of the leading processes and cell body traction during the final phase of somal translocation in the upper CP.

## Conclusion

We analyzed the effect of L1-knockdown using *in utero* electroporation of shRNA targeting L1 in murine corticogenesis. Time-lapse analyses revealed that L1-KD neurons showed decreased locomotion velocity and affected terminal translocation with aberrant leading processes and somal movement. Although the underlying molecular mechanisms remain unsolved, L1 plays an important role not only in axonal guidance, but also in neuronal migration during neocortical development.

## Supporting Information

Figure S1 **Neuro2a at 24h post-electroporation.** Both shRNA2 and shRNA5 efficiently downregulated L1cam mRNA.
(TIF)

Figure S2 **Both shRNAs downregulated L1cam at the protein level in Neuro2a.** Western blot analyses revealed that both shRNA2 and shRNA5 efficiently downregulated L1cam protein in Neuro2a.
(TIF)

Figure S3 **Cell surface L1cam was efficiently downregulated in Neuro2a.** The expression of L1cam was significantly reduced on the cell membrane of Neuro2a cells transfected by shRNA2 or shRNA5.
(TIF)

Figure S4 **Radial migration of cortical neurons was disrupted by** ***in utero*** **electroporation of shRNA5 as well as shRNA2.**
(TIF)

Text S1 **Materials and Methods of supporting experiments and Figure Legends of supporting figures and movies are described.**
(DOCX)

Movie S1 **This movie shows the time-lapse transition of migrating neurons expressing control-shRNA (shNC) from the intermediate zone (IZ) into the cortical plate (CP).** The neurons with a single leading process migrate quickly in the IZ.
(AVI)

Movie S2 **This movie shows the time-lapse transition of migrating neurons expressing L1cam-shRNA (shL1) from the intermediate zone (IZ) into the cortical plate (CP).** The neurons migrate slowly and the somas often stay in the IZ.
(AVI)

Movie S3 **This movie shows the time-lapse transition of migrating neurons expressing control-shRNA (shNC) in the cortical plate (CP).** The leading processes of the control neurons are straight and shortened in a smooth process along with somal translocation.
(AVI)

Movie S4 **This movie shows the time-lapse transition of migrating neurons expressing L1cam-shRNA (shL1) in the cortical plate (CP).** The L1-KD neurons migrate with undulated long leading processes and some of the neurons transiently cease migration.
(AVI)
