# Supplementary figures and images for: L1cam Is Crucial for Cell Locomotion and Terminal Translocation of the Soma in Radial Migration during Murine Corticogenesis
Source: PLoS One. 2014 Jan 28;9(1):e86186. doi: 10.1371/journal.pone.0086186 (PMC3904877; doi:10.1371/journal.pone.0086186)

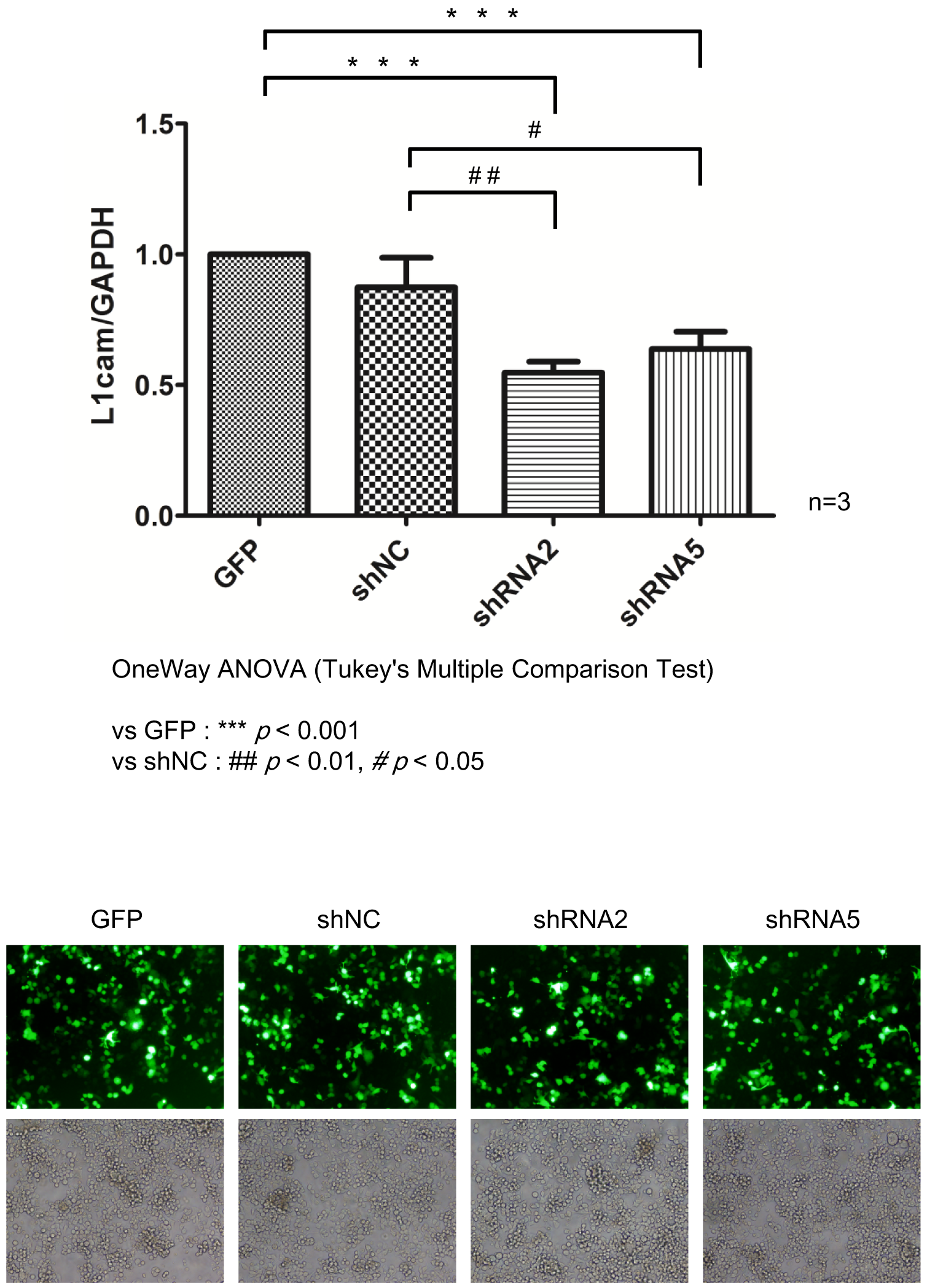

Supplement: Figure S1 — Neuro2a at 24h post-electroporation. Both shRNA2 and shRNA5 efficiently downregulated L1cam mRNA. (TIF) [file pone.0086186.s001.tif]

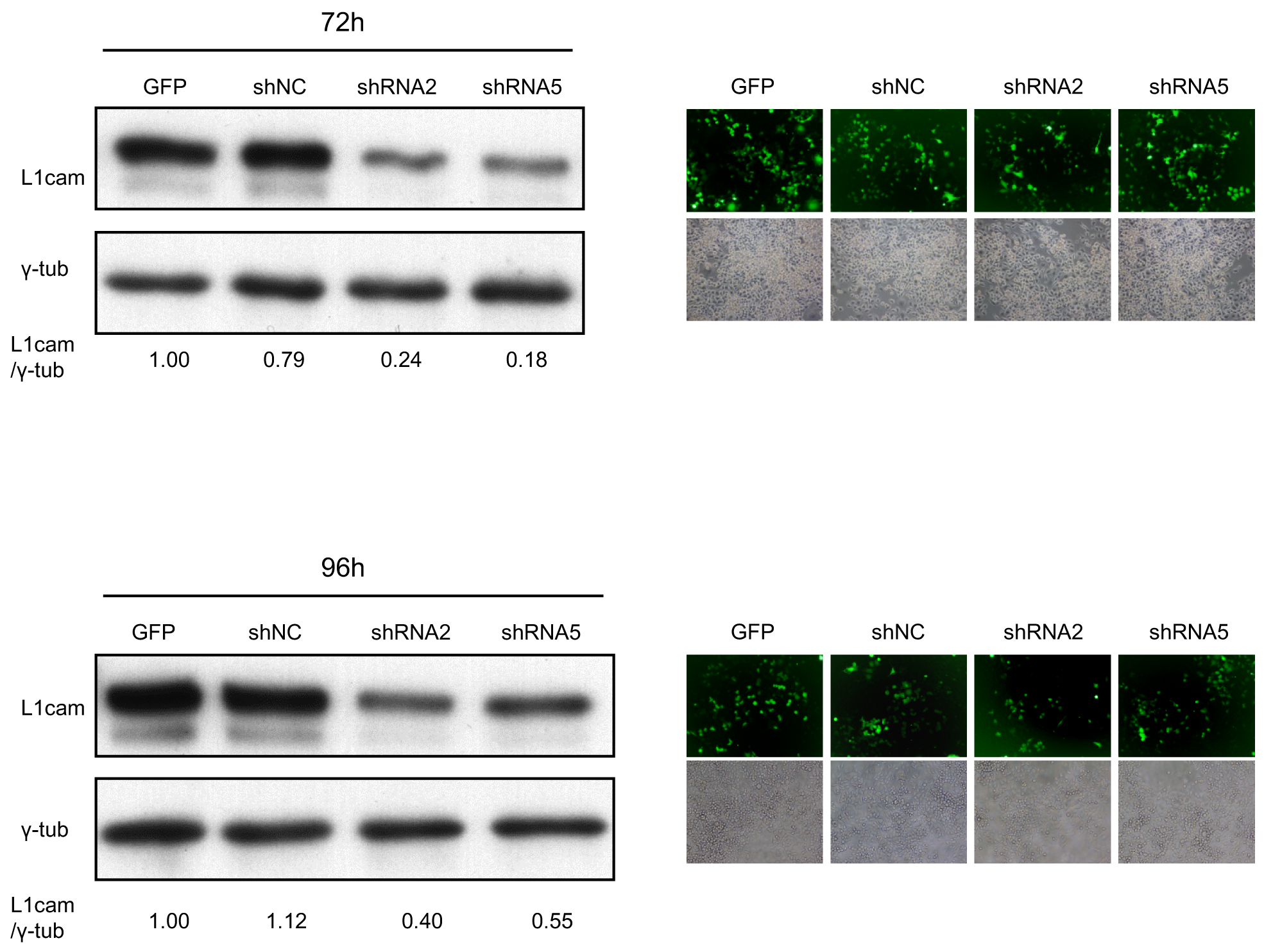

Supplement: Figure S2 — Both shRNAs downregulated L1cam at the protein level in Neuro2a. Western blot analyses revealed that both shRNA2 and shRNA5 efficiently downregulated L1cam protein in Neuro2a. (TIF) [file pone.0086186.s002.tif]

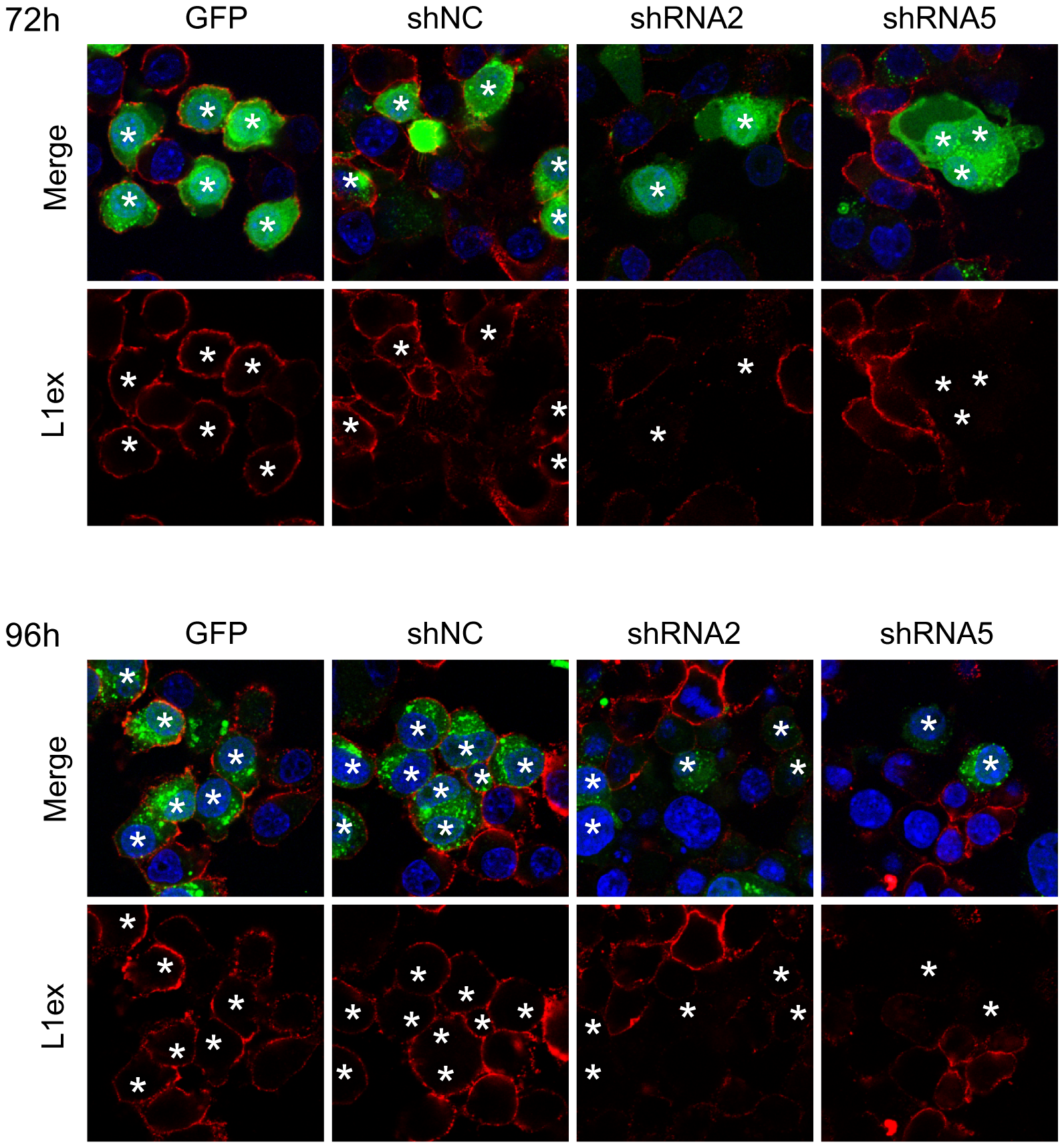

Supplement: Figure S3 — Cell surface L1cam was efficiently downregulated in Neuro2a. The expression of L1cam was significantly reduced on the cell membrane of Neuro2a cells transfected by shRNA2 or shRNA5. (TIF) [file pone.0086186.s003.tif]

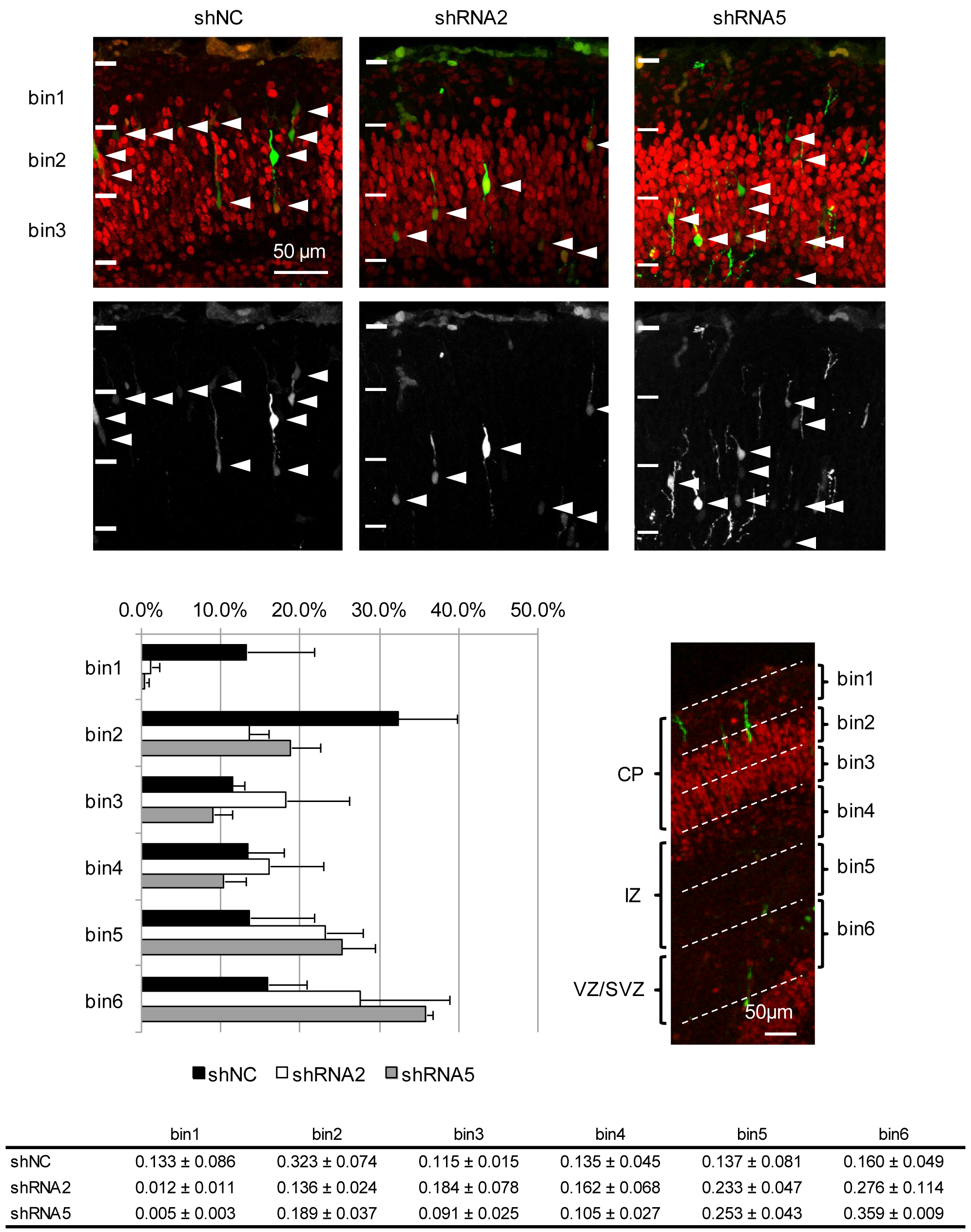

Supplement: Figure S4 — Radial migration of cortical neurons was disrupted by in utero electroporation of shRNA5 as well as shRNA2. (TIF) [file pone.0086186.s004.tif]
