# Supplementary material for: L1cam Is Crucial for Cell Locomotion and Terminal Translocation of the Soma in Radial Migration during Murine Corticogenesis
Source: PLoS One. 2014 Jan 28;9(1):e86186. doi: 10.1371/journal.pone.0086186 (PMC3904877; doi:10.1371/journal.pone.0086186)
Supplement: Text S1 — Materials and Methods of supporting experiments and Figure Legends of supporting figures and movies are described. (DOCX) [file pone.0086186.s005.docx]

*(Supplementary materials)*

Materials and Methods, Reference

Figure Legends

Figures: S1, S2, S3, S4

Movies: S1, S2, S3, S4

**Materials and Methods**

*1. Short-hairpin RNA(shRNA) Constructs and Plasmid Vectors*

We prepared two shRNAs targeting L1 cloned in a pGeneClip hMGFP vector and a negative control shRNA (shNC), as well as a hMGFP-labeled mock plasmid (GFP only plasmid). The shRNA2 was provided by SuperArray Bioscience Corporation (Frederick, MD), as previously reported, and the shRNA5 was designed and constructed in our laboratory. The siRNA sequences were as follows;

shRNA2: 5’-AGCCTTACCAGAAGGGAAAGT-3’,

shRNA5: 5’-GTGCTTCAGGATGAACGATTT-3’

shNC: 5’-GGAATCTCATTCGATGCATAC-3’.

*2. Quantitative RT-PCR*

Total RNA was extracted at 24 hours after the transfection and purified with NucleoSpin XS (Macherey-Nagel, Japan) and a TURBO DNA-free kit (Ambion, Japan). First-strand cDNAs for qRT-PCR were synthesized and amplified using a SuperScript VILO cDNA Synthesis Kit (Life Technologies Corporation, Japan) with random hexamers. The relative gene expression levels of L1 were measured by qRT-PCR on a StepOne Plus (Applied Biosystems, Japan) using a FastStart Universal SYBR Green Master (ROX) (Roche, Japan), according to the manufacturer’s protocol. The optimized number for thermal cycling was set at 40. The primers for L1 were designed as follows: 5’-CCAAGTGGAGTTCCGCTGGACG-3’ (forward) and 5’-CTTCGGCCACTTGGGGGCAC-3’ (reverse).

Due to normalization of the Ct values for L1, housekeeping genes (GAPDH) primers (Takara, Japan) were used;

5’-TGTGTCCGTCGTGGATCTGA-3’ (forward) and 5’-TTGCTGTTGAAGTCGCAGGAG-3’ (reverse).

*3. Western blotting*

Transfected cells were solubilized in RIPA buffer (50 mM Tris-HCl pH 7.5, 150 mM sodium chloride, 1% NP-40, 0.5% sodium deoxycholate, 0.1% sodium dodecyl sulfate and 5 mM EDTA) at 72 and 96 hours after the transfection. The lysate was incubated at 4°C for another 10 minutes for perfect solubilization. The lysate was centrifuged at 14,000 rpm at 4°C for 20 minutes, and the supernatant was used as a protein sample. Protein quantification was performed using a BCA Protein Assay (Thermo Fisher Scientific, Japan). Next, the protein was prepared by mixing it with SDS sample buffer, including β-mercaptoethanol, and then denatured by heating at 95-97°C for 5 minutes. The proteins were separated on a 8% polyacrylamide-SDS gel and transferred to a polyvinylidene fluoride (PVDF) membrane (0.45 μm, Millipore). After blocking in 5% skim milk / Tris Buffered Saline with 0.05% Tween 20 (TBST) at room temperature for 1 hour, the membrane was incubated in the primary antibodies (anti-L1 rabbit antibodies; 1:5,000, anti-γ tubulin mouse antibodies; 1:5,000) overnight at 4°C and finally, the anti-rabbit or anti-mouse secondary antibodies were conjugated HRP at room temperature for 1 hour. The membrane was developed for 1 minute using the enhanced chemiluminescence detection system (TAKARA Bio, Japan) and protein bands were captured on X-ray film.

*4. Immnocytochemistry (ICC)*

At 72 hours and 96 hours after the transfection, the cells were fixed with 4% paraformaldehyde (PFA) / 0.1M phosphate buffer (PB; pH 7.4) for 15 minutes at 4°C, followed by washing in phosphate-buffered saline (PBS) without detergent. After blocking in 10% (v/v) goat serum and 1% (w/v) bovine serum albumin (BSA) and phosphate-buffered saline (PBS) at room temperature for 1 hour, the cells were incubated with primary antibodies L1ex (1:5,000; gift from V. Lemmon (ref 1)) diluted in 1% (v/v) goat serum and 0.1% (w/v) BSA and PBS overnight at 4°C, followed by washing in cold PBS. The cells were incubated with secondary antibodies (Alexa647 conjugated anti-rat IgG (Molecular probe, 1:1,000)) at room temperature for 2 hours, followed by washing in cold PBS. For nuclear staining, the cells were incubated in DAPI solution (1.0 μg/ml, PBS) at room temperature for 10–15 minutes, followed by washing in cold PBS. Finally the stained cells were mounted using antifade reagent (SlowFade; invitrogen) for observation with confocal laser-microscopy (LSM 510 Meta, Carl Zeiss). Images were obtained with a Zeiss (LSM 510 Meta) microscope with the same laser intensity and adjusted for color and contrast using the LSM Image Browser.

*5. Immunohistochemistry (IHC)*

After *in utero* electroporation at 72 hours, the fetal brains were dissected and fixed overnight with 4% paraformaldehyde / 0.1M PB, followed by cryoprotection in 30% sucrose at 4°C. Fixed brains were embedded in CMC compound and frozen in powdered dry ice. Serial coronal sections, at a thickness of 20 μm, were made using a cryomicrotome (Leica CM1850). After blocking with 10% goat serum, 1% bovine serum albumin (BSA), and 0.01% Triton X-100 (Sigma-Aldrich Japan) in 0.1 M PB, the sections were incubated in primary antibody at 4°C overnight; Ctip2 (rat monoclonal, ab18465; Abcam; 1:1,000) diluted in 1% (v/v) goat serum, and 0.1% (w/v) BSA, 0.01% Triton X-100 and PBS. The sections were rinsed several times with PBS and then incubated in the secondary antibody (Alexa 647-conjugated goat anti-rat IgG, 1:1,000, Invitrogen Japan). The sections were then mounted using an antifade reagent (SlowFade; invitrogen) for observation with confocal laser-microscopy (LSM 510 Meta, Carl Zeiss). Images were obtained with a Zeiss (LSM 510 Meta) microscope and adjusted for color and contrast using the LSM Image Browser.

*6. Image Analysis*

Images were obtained with a Zeiss (LSM 510 Meta) microscope and analyzed with the ImageJ plugin Cell counter. The wall of the embryonic dorsal brain was divided into six layers; the upper (bin1), middle (bin2) and lower (bin3) cortical plate, upper (bin4) and lower (bin5) intermediate zone, and ventricular/subventricular zone (bin6). The way in which the IZ or the CP was divided into two or three subzones, respectively, was simply by dividing the region into two or three equal parts. The total numbers of MGFP-expressing transfected cells were counted in each of the six layers in each brain, and the ratio (percentage) of the cells present in each layer was calculated. We counted a total of 269 cells (n = 2), 421 cells (n = 2), and 1,169 cells (n = 4), from shNC (E13–16), shRNA2 (E13–16), and shRNA5 (E13–16), respectively.

*7. Statistical analysis*

To calculate statistical significance, One-way ANOVA followed by post hoc Tukey-Kramer using GraphPad Prism software was performed. The data were shown as the mean ± standard error of the mean (SEM), and *P* < 0.05 was considered to be statistically significant.

*8. References*

1. Nakamura Y, Lee S, Haddox CL, Weaver EJ, Lemmon VP (2010) Role of the cytoplasmic domain of the L1 cell adhesion molecule in brain development. J Comp Neurol 518:1113-1132

**Figure Legends**

**Figure S1. Neuro2a at 24h post-electroporation**

Both shRNA2 and shRNA5 efficiently downregulated L1cam mRNA as compared to shNC and GFP (GFP-expressing vector without shRNA) in Neuro2a at 24 hours after the transfection. The transfection efficiency was similar in shRNA2, shRNA5, shNC and in GFP.

**Figure S2. Both shRNAs downregulated L1cam at the protein level in Neuro2a**

Western blot analyses revealed that both shRNA2 and shRNA5 efficiently downregulated L1cam protein in Neuro2a at 72 hours and 96 hours after the transfection.

The transfection efficiency was similar in shRNA2, shRNA5, shNC and in GFP (right panel).

**Figure S3. Cell surface L1cam was efficiently downregulated in Neuro2a**

The expression of L1cam was significantly reduced on the cell membrane of Neuro2a cells transfected by shRNA2 or shRNA5, as compared with shNC and GFP at 72 hours (upper panel) and 96 hours (lower panel) after the transfection. * indicates a shRNA- transfected cell.

**Figure S4. Radial migration of cortical neurons was disrupted by *in utero* electroporation of shRNA5 as well as shRNA2.**

In shRNA2 or shRNA5-transfected cortical neurons, more neurons were detected in the bin5 and bin6, whereas fewer neurons reached bin1 and bin2 as compared with shNC-transfected neurons.

**Movie S1**

This movie shows the time-lapse transition of migrating neurons expressing control-shRNA (shNC) from the intermediate zone (IZ) into the cortical plate (CP). 　The neurons with a single leading process migrate quickly in the IZ. The slice prepared at E14.5, one day after the *in utero* electroporation, was observed from Div2 every 10 minutes for 12 hours.

**Movie S2**

This movie shows the time-lapse transition of migrating neurons expressing L1cam-shRNA (shL1) from the intermediate zone (IZ) into the cortical plate (CP). The neurons migrate slowly and the somas often stay in the IZ. The slice prepared at E14.5, one day after the *in utero* electroporation, was observed from Div2 every 10 minutes for 12 hours.

**Movie S3**

This movie shows the time-lapse transition of migrating neurons expressing control-shRNA (shNC) in the cortical plate (CP). The leading processes of the control neurons are straight and shortened in a smooth process along with somal translocation. The black line demonstrates the pia mater. The slice prepared at E15.5, two days after the *in utero* electroporation, was observed every 10 minutes for 7 hours.

**Movie S4**

This movie shows the time-lapse transition of migrating neurons expressing L1cam-shRNA (shL1) in the cortical plate (CP). The L1-KD neurons migrate with undulated long leading processes and some of the neurons transiently cease migration. L1-KD neuron does not finish terminal translocation within the observation period and settles in aberrant position in the deeper CP with tangentially-directed leading process. The slice prepared at E15.5, two days after the *in utero* electroporation, was observed every 10 minutes for 12 hours.
